# Productive herpesvirus lytic replication in primary effusion lymphoma cells requires S-phase entry

**DOI:** 10.1099/jgv.0.001444

**Published:** 2020-06-05

**Authors:** Robert Hollingworth, Grant S. Stewart, Roger J. Grand

**Affiliations:** ^1^​ Institute of Cancer and Genomic Sciences, University of Birmingham, Birmingham, UK

**Keywords:** KSHV, HHV-8, herpesvirus, cell cycle, S phase, lytic replication, replication stress, DNA damage response, palbociclib

## Abstract

Gammaherpesviruses establish lifelong latent infection in B lymphocytes and are the causative agent of several B-cell malignancies and lymphoproliferative disorders. While a quiescent latent infection allows these pathogens to evade immune detection, initiation of an alternative lifecycle stage, known as lytic replication, is an essential step in the production and dissemination of infectious progeny. Although cessation of cellular proliferation is an eventual consequence of lytic induction, exactly how gammaherpesviruses manipulate the cell cycle prior to amplification of viral DNA remains under debate. Here we show that the onset of Kaposi's sarcoma-associated herpesvirus (KSHV) lytic reactivation in B cells leads to S-phase accumulation and that exit from G1 is required for efficient viral DNA replication. We also show that lytic replication leads to an S-phase-specific activation of the DNA damage response (DDR) that is abrogated when lytic replication is restricted to G0/G1. Finally, we observe that expression of early lytic viral genes results in cellular replication stress with increased stalling of DNA replication forks. Overall, we demonstrate that S-phase entry is important for optimal KSHV replication, that G1 arresting compounds are effective inhibitors of viral propagation, and that lytic-induced cell-cycle arrest could occur through the obstruction of cellular replication forks and subsequent activation of the DDR.

## Introduction

Gammaherpesviruses are double-stranded DNA viruses that infect and persist in B lymphocytes and can induce their proliferation and transformation. Kaposi's sarcoma-associated herpesvirus (KSHV) is a member of the *Gammaherpesvirinae* family that is responsible for the lymphoproliferative diseases multicentric Castleman’s disease (MCD) and primary effusion lymphoma (PEL) [1, 2]. KSHV, like other herpesviruses, has two distinct lifecycle stages known as latent infection and lytic replication. Latency is established following nuclear entry and is characterized by translation of only a limited number of viral proteins that aid maintenance of extrachromosomal viral episomes while promoting host-cell survival and cell-cycle progression. Although the quiescent latency phase is advantageous for evading host-cell immune surveillance and establishing lifelong persistence, viral dissemination requires rapid amplification of viral genomes and assembly of infectious virions. To this end, herpesviruses sporadically execute a lytic replication phase that involves expression of the full repertoire of viral genes. Viral genes expressed specifically in the lytic phase have been grouped into immediate-early genes (expressed first and required for expression of other lytic genes), early genes (including those that encode viral replication proteins) and late genes (includes those that encode structural proteins required for virion assembly). Viral episomes are duplicated in globular domains in the nucleus known as replication compartments (RCs) and the concluding stages of productive lytic replication involve lysis of the host cell permitting rapid egress of infectious progeny.

DNA viruses must manipulate the host-cell cycle in order to promote efficient replication of their genetic material. Small DNA viruses, such as papillomaviruses and adenoviruses, rely on the host replication machinery for viral genome replication and consequently facilitate S-phase entry before viral DNA amplification proceeds. During latent infection, KSHV DNA is also replicated by host polymerases during S phase and these duplicated episomes are then segregated to daughter cells along with cellular DNA during mitosis. The situation regarding cell-cycle manipulation during lytic replication of gammaherpesviruses is less well-defined. As these pathogens encode their own replisome components, they are, in theory, less dependent on host replication resources for their successful propagation. Although the transition to the lytic replication phase will inevitably lead to cessation of the cell cycle, since cellular DNA replication is halted and cell lysis eventually occurs, there have been conflicting reports regarding the cell-cycle phase in which productive lytic replication takes place. Earlier reports indicated that lytic cycle initiation leads to an accumulation of cells in G1 [3, 4]. The proposed rationale was that, by restricting S-phase entry, the virus avoids competition with cellular DNA for replication resources. In support of these findings, both the KSHV immediate-early proteins RTA and K8α have been shown to negatively regulate the G1/S transition when expressed alone [4, 5]. In contrast, another study presented evidence that S phase provides a more conducive environment for KSHV replication due to upregulation of genes that promote DNA replication, cell survival and lipid metabolism [6]. More recently, the G2/M checkpoint has also been implicated as a target during KSHV lytic replication [7]. The authors showed that iSLK.219 cells, containing recombinant KSHV, bypass the G1/S checkpoint following lytic induction but accumulate in G2/M via stimulation of the p53-p21 signalling axis.

We, and others, have previously reported that KSHV lytic replication results in cellular DNA damage and concurrent activation of the DNA damage response (DDR) [8–11]. As part of our previous report into the effect of DDR kinase inhibitors on KSHV replication efficiency, we observed, through examining relative DNA content, that inducing KSHV lytic replication in a PEL line increased the proportion of these cells in S phase [10]. We also observed that the reduction in replication efficiency detected following ATR inhibition was also accompanied by an increase in the number of G1 arrested cells. Furthermore, one of the DDR-related signalling events that occurred in these cells during lytic replication was phosphorylation of RPA32 on serines 4 and 8. This phosphorylation event is associated with homologous recombination-dependent DNA end resection that occurs in S and G2 phases of the cell cycle suggesting that the DDR elicited during the lytic phase may occur outside of G1.

In this report, we have closely examined the cell-cycle changes that take place in B cells containing lytically replicating KSHV and assessed the importance of these manipulations for viral replication efficiency. Using several well-established indicators of cell-cycle phase distribution, we demonstrate here that lytic replication of KSHV in the BCBL-1 PEL cell line leads to S-phase accumulation. In addition, we show that imposing a G1 block in these cells prior to lytic induction results in significantly reduced viral replication efficiency. Finally, we show that expression of early viral genes results in cellular DNA replication stress and S-phase-specific activation of the DDR, which is abrogated when lytic replication is limited to the G0/G1 phase. Overall, we demonstrate that host transition through the G1/S-phase checkpoint is an important aspect of the KSHV lytic replication strategy and that chemical inhibition of S-phase entry is an effective means of restricting herpesvirus propagation.

## Results

### Reactivation of KSHV in PEL cells leads to S-phase accumulation

**Fig. 1.**
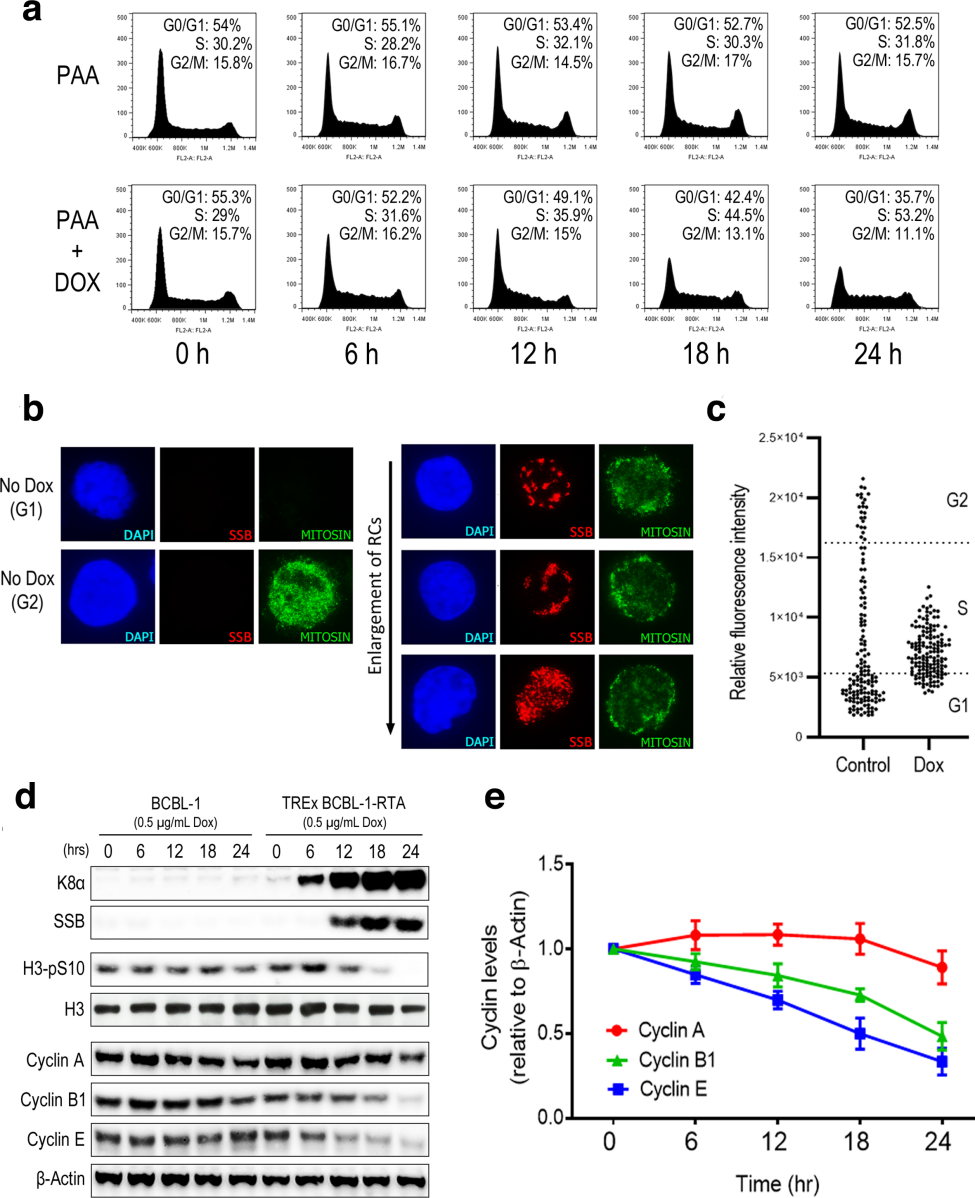
Reactivation of KSHV in PEL cells leads to S-phase accumulation. (a) Cell-cycle profiles of TREx BCBL-1-RTA cells treated with either PAA or PAA and doxycycline for the indicated times. Percentages of cell-cycle phase distribution represent the average of three independent experiments. (b) Nuclear expression of mitosin following lytic reactivation of KSHV in TREx BCBL-1-RTA cells. Included are representative images of mitosin levels in G1 and G2 as well as images of cells containing viral RCs marked by the viral SSB protein. (c) Relative mitosin intensities corrected for background in asynchronous control cells and dox-treated KSHV RC-positive cells. (d) Western-blot analysis of cyclin proteins and phospho H3 levels during the KSHV lytic replication cycle after doxycycline treatment of BCBL-1 and TREx BCBL-1-RTA cells. Expression of viral K8α and SSB confirm induction of lytic reactivation. (e) Densitometry analysis of relative levels of cyclins A, B1 and E in TREx BCBL-1-RTA cells following addition of doxycycline. Data points represent the average of three repeat experiments. Values were calculated relative to β-Actin intensity and expressed as a ratio of un-reactivated control cells. Error bars represent the standard error of the mean (sem).

BCBL-1 is a PEL cell line derived from a post-germinal centre B-cell population that contains multiple copies of the KSHV episome [12]. TREx BCBL-1-RTA cells have been modified to express the lytic switch protein RTA from a doxycycline-inducible promotor [13]. Initially, we wanted to confirm that KSHV lytic replication in B cells leads to cell-cycle arrest in S phase. We therefore employed propidium iodide (PI) staining combined with flow cytometry, a well-characterized cell-cycle assay based on relative DNA content, in order to assess what cell-cycle changes take place following induction of lytic replication (Fig. 1a). Experiments were performed using the inhibitor of viral DNA synthesis phosphonoacetic acid (PAA) in order to ensure that viral DNA was not detected as cellular DNA leading to overrepresentation of S-phase cells [14]. TREx BCBL-1-RTA cells were treated with PAA alone or PAA in combination with doxycycline and harvested at 6 h intervals up until 24 h. Cell-cycle profiles of cells containing lytic KSHV were compared with uninduced controls at the same time points. Treatment with PAA alone did not result in any notable cell-cycle changes in these cells after 24 h. In contrast, treatment with doxycycline and PAA led to a notable increase in the number of cells in S phase after 24 h with a corresponding reduction in the number of cells in G0/G1 and G2/M.

In cells containing lytic virus, KSHV RCs can be visualised with immunofluorescence microscopy (IF) using an antibody against the viral single-stranded DNA binding protein (SSB) [15]. To confirm that cells containing lytic KSHV enter S phase, we monitored levels of the cell-cycle marker protein mitosin, to determine at what phase of the cell cycle KSHV RCs form. Mitosin is a kinetochore-associating protein that accumulates gradually as cells progress through S phase and is expressed maximally in G2 and M phases before being degraded following the completion of mitosis [16]. Mitosin is a commonly used cell-cycle marker employed in IF as cells with bright nuclear mitosin staining are known to be in the G2 phase, mitosin-negative cells represent those in G1, while S-phase cells contain intermediate levels of this protein. Mitosin levels during formation of KSHV RCs were examined by IF in TREx BCBL-1-RTA cells treated with doxycycline for 24 h(Fig. 1b, c). Images of cells with mitosin staining typical of G2 and G1 cells that were negative for lytic KSHV are included for comparison (Fig. 1b). We observed that cells containing KSHV RCs displayed mitosin staining that was intermediate between that of G1 and G2 cells indicating that KSHV RCs form in S phase. Images of multiple cells containing viral RCs of differing sizes were taken to represent progressive stages of viral DNA amplification (Fig. 1b). Relative mitosin intensities in both asynchronous uninduced cells and in dox-treated viral RC-positive cells were also calculated and compared using a distribution dot plot (Fig. 1c). The cell-cycle distribution of uninduced TREx BCBL-1-RTA cells determined by PI staining was used to indicate the range of mitosin intensity values that corresponded to each cell-cycle phase. This analysis clearly illustrates that cells containing lytic KSHV have a narrower distribution of mitosin intensities compared with control cells. It was also determined that 76 % of all RC-positive cells measured had a mitosin intensity value that lies within the range that corresponds to S phase in control cells (Fig. 1c).

As an additional indicator of cell-cycle distribution during lytic replication, Western blotting was used to monitor levels of cell-cycle marker proteins following doxycycline treatment of control BCBL-1 cells and TREx BCBL-1-RTA cells (Fig. 1d). Blotting for the KSHV lytic proteins K8α and SSB was used to verify induction of viral replication in TREx BCBL-1-RTA cells. To confirm that cells containing lytic virus do not enter mitosis, levels of phosphorylated H3 at serine 10 were also examined. Levels of this mitotic marker were clearly reduced 18 h after lytic induction compared with uninduced controls indicating that lytic replication does not lead to mitotic accumulation (Fig. 1d). Next, levels of cyclin A, B1 and E were examined at the same time points (Fig. 1d, e). Cyclins are a group of proteins that regulate cell-cycle progression through interactions with cyclin-dependent kinases (CDKs) and whose expression varies throughout the cell cycle [17]. Expression of cyclin A increases during S phase and is maximal at the end of G2, cyclin B1 levels peak at the G2/M boundary, while cyclin E levels are maximal during the G1 to S-phase transition. Levels of both cyclin B1 and cyclin E were notably reduced following doxycycline treatment indicating that viral reactivation does not lead to an accumulation of cells at the G1/S or G2/M boundaries. Levels of cyclin A remained relatively stable but appeared slightly reduced by 24 h. Since cyclin A levels are low at the start of S phase and accumulate gradually until G2, a slight reduction in this cyclin during the lytic cycle could be due to cells arresting in early to mid-S phase. Western blots of these three cyclin proteins were also quantified relative to β-Actin across three repeat experiments using densitometry (Fig. 1e). This analysis clearly shows a consistent reduction in cyclins B1 and E during lytic replication in contrast to the relatively steady levels of cyclin A. Overall, these findings demonstrate that induction of lytic replication in BCBL-1 cells leads to cell-cycle arrest in S phase where formation and expansion of viral RCs takes place.

### Restricting S-phase entry in PEL cells using serum depletion attenuates KSHV replication and DDR activation following lytic induction

**Fig. 2.**
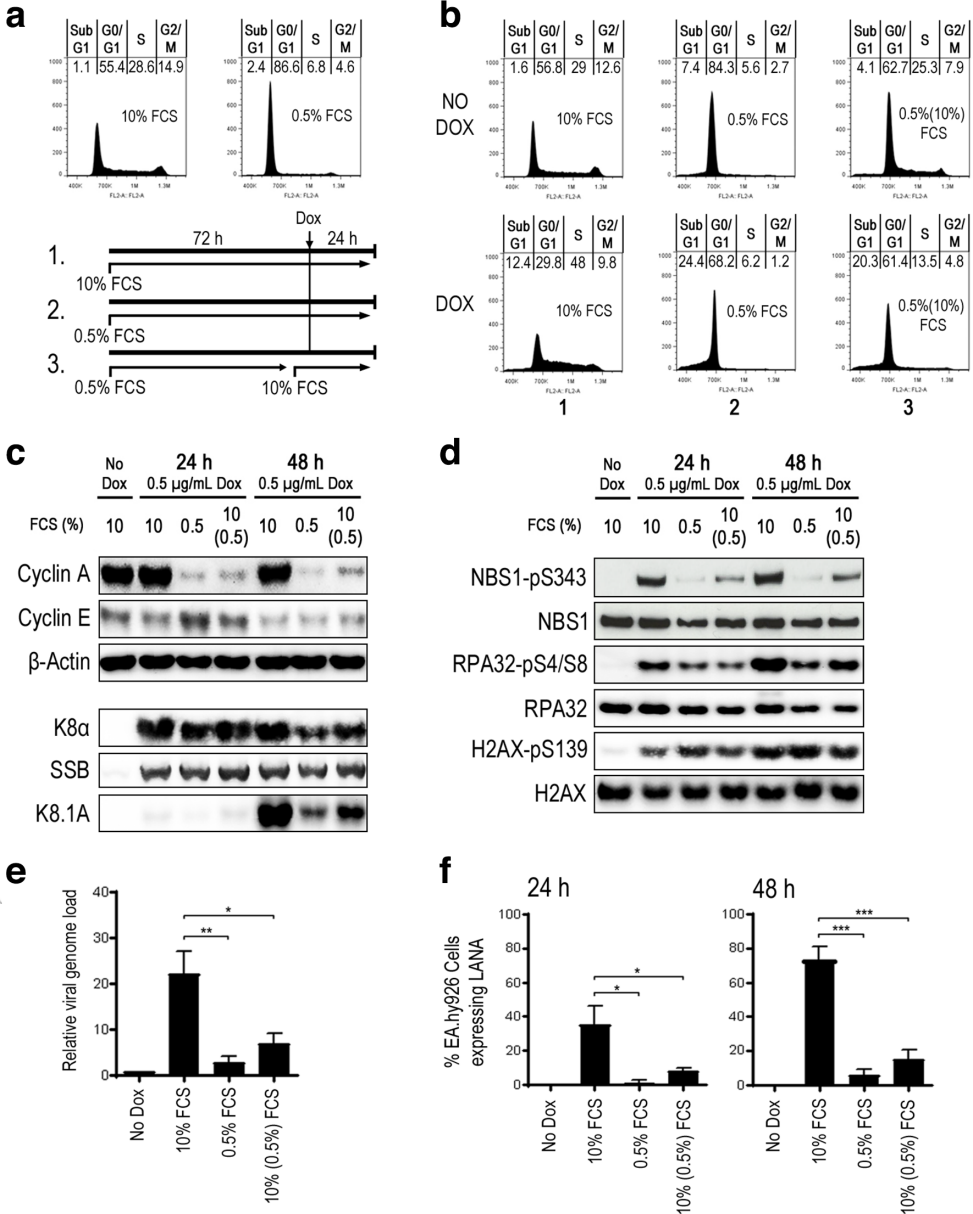
Restricting S-phase entry in PEL cells using serum depletion attenuates KSHV replication and DDR activation following lytic induction. (a) Representative cell-cycle distribution of TREx BCBL-1-RTA cells following 72 h culture in either 10 % or 0.5 % serum. (b) Representative cell-cycle profiles 24 h following doxycycline treatment in TREx BCBL-1-RTA cells cultured in normal serum (10 % FCS), serum depleted (0.5 % FCS) or serum-replenished conditions (0.5 % FCS followed by 10 % FCS). Percentages of cell-cycle phase distribution represent the average of three independent experiments. (c, d) Western-blot analysis of induced TREx BCBL-1-RTA cells cultured in the above serum concentrations measuring levels of (c) cyclins A and E and lytic proteins K8α, SSB and K8.1A as well as (d) total and phosphorylated DDR proteins NBS1, RPA32 and H2AX. (e, f) Lytic replication efficiency in induced TREx BCBL-1-RTA cells cultured in the above serum concentrations measured by (e) qPCR analysis of relative genome load 48 h after lytic induction and (f) IF quantification of release of infection virus 24 and 48 h following doxycycline treatment. Statistical analyses were performed using a two-tailed and unpaired Student *t*-test, *, *P*<0.05; **, *P*<0.01; ***, *P*<0.001. Error bars represent the sem.

Having determined that lytic replication leads to S-phase accumulation in B cells, we next wanted to assess whether restriction of S-phase entry has a negative effect on viral replication efficiency. We initially used serum depletion to induce a G0/G1 block in TREx BCBL-1-RTA cells prior to viral reactivation. By varying depletion times and serum levels, we found that reducing the serum concentration to 0.5 % for 72 h resulted in between 80 and 90 % of cells restricted to the G0/G1 phase and only a minimal increase in the sub-G1 fraction (<1.5 %) representing apoptotic cells (Fig. 2a).

In order to test the effect of serum depletion on KSHV lytic replication, TREx BCBL-1-RTA cells were cultured with 10 % or 0.5 % serum for 72 h before the addition of doxycycline. In addition, a third population of cells were cultured in 0.5 % serum for 72 h before being supplemented with 10 % serum 2 h prior to doxycycline treatment (see schematic Fig. 2a). Initially, cells were harvested after 24 h and cell-cycle profiles were compared with uninduced cells (Fig. 2b). In the absence of doxycycline, the cells depleted of serum for 96 h had predominantly accumulated in G0/G1 with 5.6 % in S phase on average compared with a mean of 29 % in the cells with normal serum levels. Cells with serum restored 72 h after serum depletion did not appear to be synchronized 24 h later and the G0/G1 fraction remained elevated compared with control cells. This condition was included here as it provides a control for lytic replication in the presence of normal serum levels but with restricted exit from G0/G1. Following addition of doxycycline to asynchronous cells, there was an increase in the S-phase fraction and a reduction in the G0/G1 and G2/M fractions as previously shown. In contrast, serum-depleted cells treated with doxycycline remained primarily in G0/G1 with an accompanying increase in the sub-G1 fraction indicating elevated cell death. Restoration of serum prior to lytic induction resulted in more than twice the proportion of cells in S phase compared with serum depleted cells although the majority were still in G0/G1 and the sub-G1 fraction was also elevated compared with the asynchronous controls.

Western blotting was also used to determine whether expression of lytic viral genes was affected by serum depletion (Fig. 2c). Levels of cyclin A and E were also monitored to confirm the restriction of S-phase entry. Levels of the immediate early protein K8α and the early viral SSB protein were unaffected by these culture conditions although levels of the late K8.1A, which is expressed after amplification of viral DNA, were clearly reduced following serum depletion. In addition, activation of the DDR following lytic induction was also assessed by blotting for levels of phosphorylated NBS1, RPA32 and H2AX (Fig. 2d). Restriction of S-phase entry resulted in a reduction in phosphorylation of RPA32 at serines 4 and 8 and NBS1 at serine 343 although the level of phosphorylated H2AX remained elevated.

To determine if these culture conditions resulted in reduced viral replication efficiency, relative viral genome load was determined by qPCR 48 h following doxycycline treatment (Fig. 2e). In serum-depleted cells there was a sharp reduction in viral genome amplification, which was only partially rescued in the serum-restoration conditions. The relative reduction in viral DNA replication efficiency in these culture conditions was approximately proportional to the fraction of cells entering S phase 24 h following doxycycline treatment. Production of infectious virus following serum depletion after both 24 and 48 h was also determined by treating EA.hy926 cells with supernatants from induced TREx BCBL-1-RTA cells and quantifying the number of infected cells by immunofluorescence staining for the latent KSHV protein LANA (Fig. 2f). There was a similar reduction in infectious virus production in both serum-depleted conditions after both 24 and 48 h confirming the attenuation of viral replication following restricted S-phase entry. Overall, these results indicate that S-phase entry is required for proficient KSHV replication and activation of specific aspects of the DDR during the lytic cycle.

### Treatment of PEL cells with the CDK4/6 inhibitor palbociclib results in G1 accumulation and reduced viral replication efficiency

**Fig. 3.**
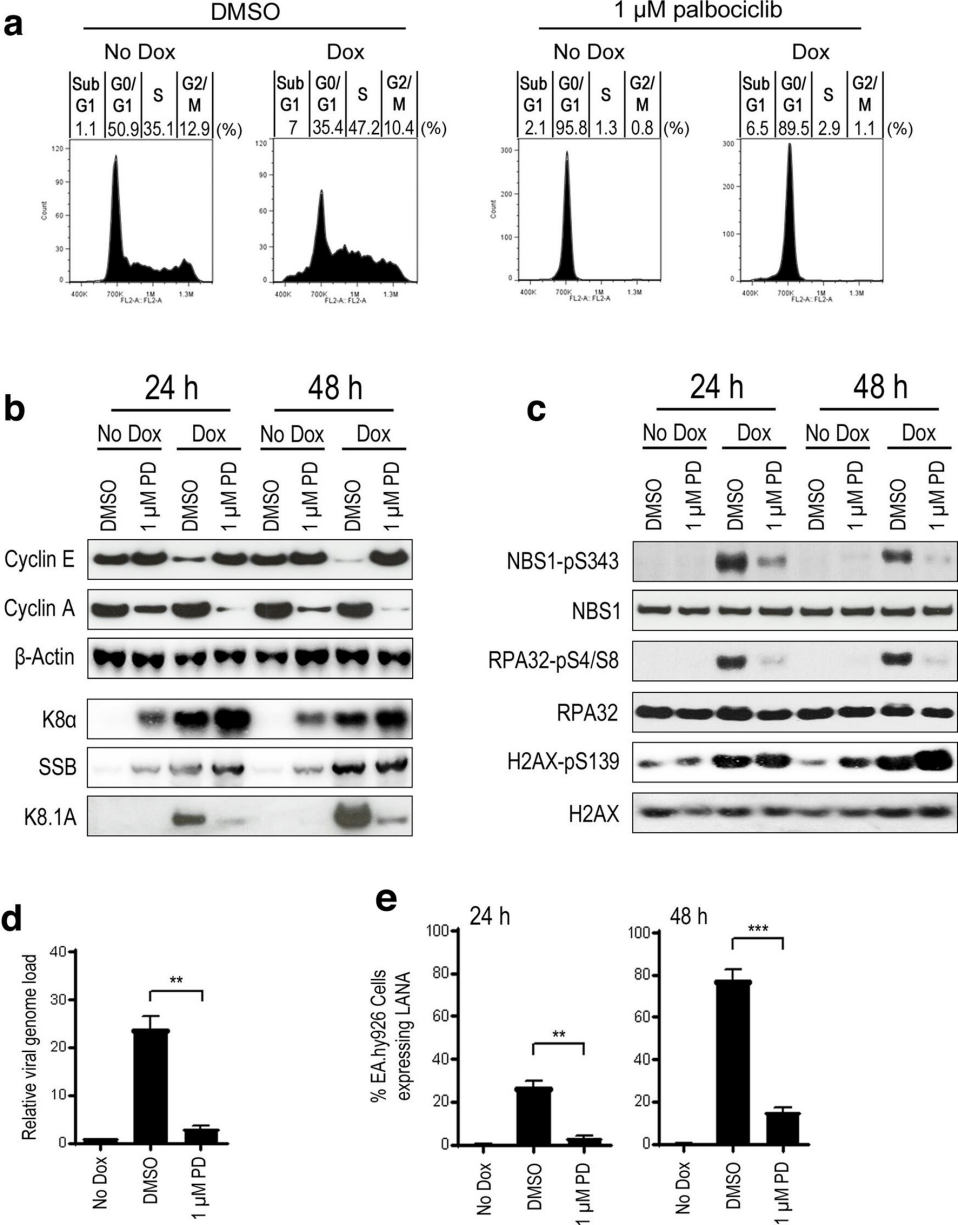
Treatment of PEL cells with the CDK4/6 inhibitor palbociclib results in G1 accumulation and reduced viral replication efficiency. (a) Representative cell-cycle profiles of TREx BCBL-1-RTA cells treated with either DMSO or palbociclib for 1 h followed by doxycycline for 24 h. Percentages of cell-cycle phase distribution represent the average of three independent experiments. (b, c) Western-blot analysis of induced TREx BCBL-1-RTA cells treated with either palbociclib or DMSO measuring levels of (b) cyclins A and E and lytic proteins K8α, SSB and K8.1A as well as (c) total and phosphorylated DDR proteins NBS1, RPA32 and H2AX. (d, e) Lytic replication efficiency in induced TREx BCBL-1-RTA cells treated with either palbociclib or DMSO measured by (d) qPCR analysis of relative genome load 48 h after lytic induction and (e) IF quantification of release of infection virus 24 and 48 h following doxycycline treatment. Statistical analyses were performed using a two-tailed and unpaired Student *t*-test, **, *P*<0.01; ***, *P*<0.001. Error bars represent the sem.

To confirm that restriction of S-phase entry has a negative effect on viral replication, we next employed a reversible small-molecule inhibitor of CDK4/6 known as palbociclib that induces a G1 phase arrest in proliferating cells [18, 19]. In the absence of doxycycline, addition of 1 µM palbociclib to TREx BCBL-1-RTA cells caused over 95 % to accumulate in G0/G1 after 24 h (Fig. 3a). Induction of lytic replication using doxycycline 1 h following palbociclib treatment resulted in an average of less than 3 % of cells entering S phase after 24 h compared with an average of 47 % in cells treated with DMSO. In addition, the presence of palbociclib during lytic replication did not lead to an increase in the proportion of cells in the sub-G1 fraction that typically represents apoptotic cells.

Having established that this compound restricts S-phase entry during lytic replication in TREx BCBL-1-RTA cells, we next examined palbociclib-induced changes in lytic gene expression and DDR activation by Western blotting (Fig. 3b, c). As before, expression of cyclin A and E was used to confirm G1 accumulation following palbociclib treatment while levels of the immediate-early K8α, early SSB and late K8.1A viral proteins were examined to evaluate lytic gene expression in G1 (Fig. 3b). Following doxycycline treatment, levels of immediate early K8α and early SSB were actually elevated in G1 arrested cells but expression of late K8.1A was severely attenuated compared with the DMSO control suggesting restricted amplification of viral DNA. It was also notable that, in the absence of doxycycline, CDK4/6 inhibition resulted in increased levels of immediate early K8α and early SSB but not late K8.1A. This suggests that this compound alone promotes initiation of the early lytic gene cascade but that this does not progress as far as expression of late viral genes. Similarly to the observation in serum-depleted cells, inhibition of S-phase entry with palbociclib during KSHV lytic replication ablated phosphorylation of the DDR substrates NBS1 and RPA32 (Fig. 3c). As also seen in serum-depleted cells, levels of phosphorylated H2AX were not reduced in G1-arrested cells but were in fact elevated after 48 h.

Next, we wanted to confirm that palbociclib had a negative effect on KSHV lytic replication efficiency as indicated by the reduction in levels of K8.1A following G1 arrest. Multiplication of viral genomes was first measured by qPCR and calculated relative to latently infected cells (Fig. 3d). Inhibition of CDK4/6 drastically reduced viral genome load 48 h after initiation of lytic replication compared with vehicle-treated controls. As before, infectious virus release was also determined my measuring infectivity of viral supernatants following infection of endothelial cells (Fig. 3e). Treatment with palbociclib prior to doxycycline resulted in a reduction in the release of infectious virus after 24 and 48 h compared with DMSO-treated controls. Treatment with palbociclib alone did not increase the percentage of infected cells compared with uninduced, DMSO-treated controls. Similar to the findings using serum depletion, these results confirm that restriction of S-phase entry in PEL cells restricts KSHV replication and attenuates activation of the DDR following initiation of the lytic programme.

### Expression of early lytic genes leads to increased cellular DNA fork stalling

Activation of the DDR in S phase can be the result of DNA replication stress, defined as any event that interferes with the progress of the DNA replication machinery. To see if S-phase arrest and DDR activation during KSHV lytic replication is accompanied by DNA replication stress, we employed the DNA fibre assay to determine the relative occurrence of replication fork stalling in cells containing lytic KSHV. This assay involves sequential labelling of DNA with two thymidine analogues, chloro-deoxyuridine (CldU) and iodo-deoxyuridine (IdU), that are fluorescently labelled following spreading of the DNA on glass slides. The DNA structures generated can then be imaged and quantified as ongoing replication forks, containing both CldU and IdU, or forks that have stalled in the first label and therefore contain only CldU. While it is possible that replication forks will stall in the second label leading to shorter IdU tracts compared with CldU, shorter IdU tracts could also be the result of slowing of replication and are therefore not classified as stalled forks here.

**Fig. 4.**
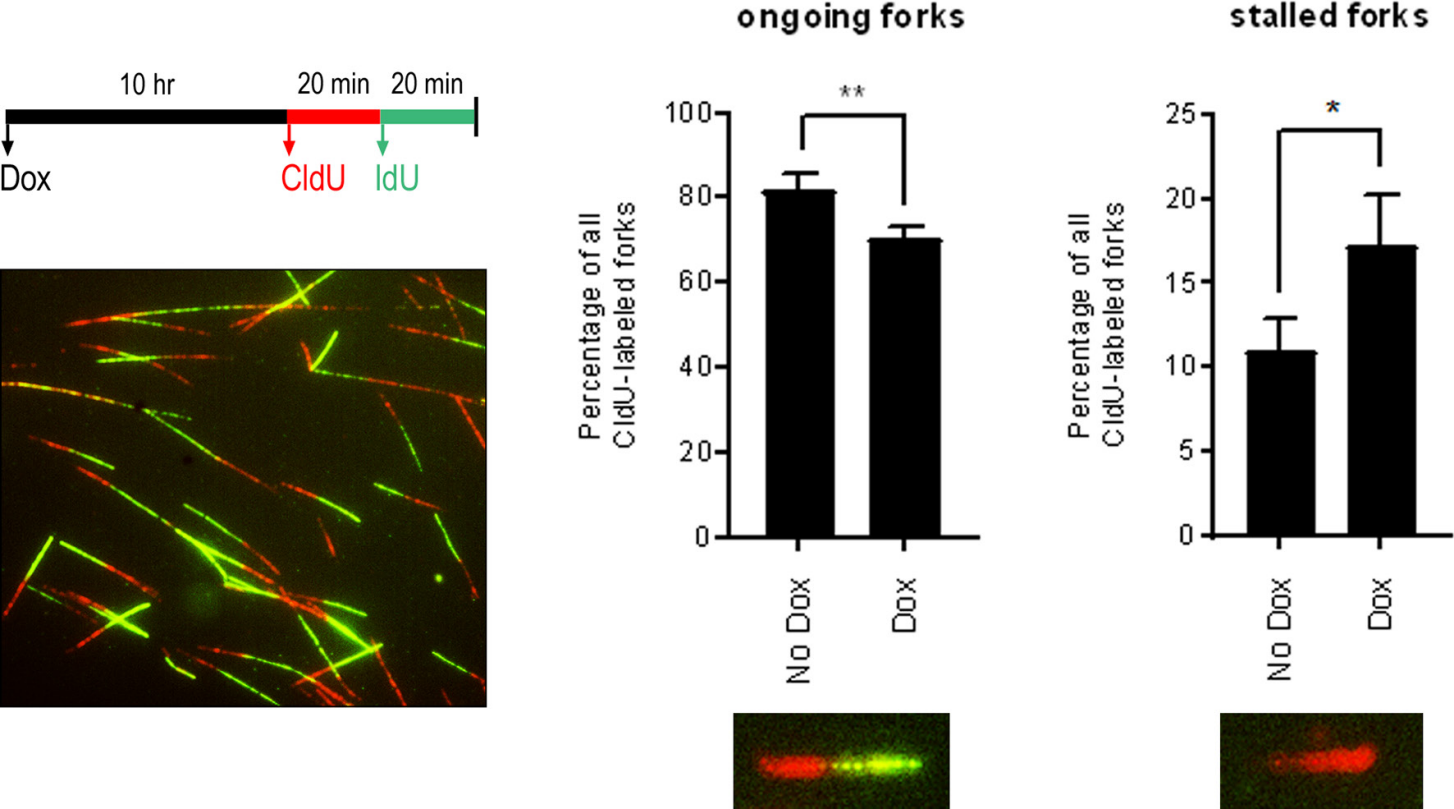
Expression of early lytic genes leads to increased cellular DNA fork stalling. DNA fibre analysis of ongoing and stalled replication forks in TREx BCBL-1-RTA cells treated with doxycycline for 10 h. Cells were pulsed with CldU for 20 mins followed by IdU for 20 mins before DNA spreading and immunofluorescence staining. A minimum of 500 DNA fibres were analysed for each of three repeat experiments. Statistical analyses were performed using a two-tailed and unpaired Student *t*-test, *P<0.05; **, *P*<0.01. Error bars represent the sem.

TREx BCBL-1-RTA cells were treated with doxycycline for 10 h before being pulse labelled with the nucleoside analogues CldU and IdU for 20 mins each (Fig. 4). Replication was analysed at 10 h after doxycycline treatment as this is sufficient time for expression of immediate-early and early viral genes but prior to amplification of viral genomes, which minimizes the possibility of assaying viral, rather than cellular, DNA [20]. This DNA fibre analysis revealed that induction of lytic replication resulted in a significant decrease in the percentage of ongoing replication forks (CldU+IdU), and an accompanying increase in the proportion of stalled replication forks (CldU only) compared with latently infected PEL cells. This observation indicates that early lytic gene expression leads to cellular DNA replication stress, which impedes replication fork progression.

## Discussion

Here we have shown that S-phase entry occurs following expression of KSHV lytic genes and is a prerequisite for optimal viral replication in PEL cells. We cannot rule out that viral proteins have a direct regulatory effect on some of the host cell cycle proteins examined here. However, a higher DNA content in the absence of viral replication, positive staining for mitosin and reduced levels of cyclin E all indicate that B cells containing lytic KSHV do not accumulate in G1 or at the G1/S transition. These findings are in contrast to earlier reports proposing that avoidance of S-phase entry is a specific replication strategy employed by several herpesviruses including KSHV, Epstein-Barr virus (EBV) and herpes simplex virus 1 (HSV-1) [4, 21, 22]. However, in support of our finding that KSHV lytic replication occurs in S phase, a previous study using EBV-infected epithelial cells has demonstrated that the replication factories of this closely related gammaherpesvirus also form outside of G1 [23]. Using a Cdt1-eGFP fusion protein to track cell-cycle progression in live cells containing lytic virus, the authors observed that EBV RCs develop in early S phase and sequester the PCNA DNA clamp while excluding cellular DNA and histones. Previous studies demonstrating that individual expression of the KSHV lytic proteins RTA and K8α results in G1 arrest have provided additional evidence that prevention of S-phase entry is an advantageous herpesvirus adaptation [4, 5]. However, it is also known that V-cyclin, a KSHV d-type cyclin homologue expressed in both the latent and lytic phases, inactivates p27 promoting cell-cycle progression through G1 into S phase [24]. The phosphorylation of retinoblastoma protein (RB) by herpesvirus-encoded kinases has also been demonstrated to promote transit through the G1/S transition [25]. Overall, while potentially informative, cell-cycle manipulations observed following expression of individual viral genes should be considered in the context of the wider lytic expression cascade.

To examine the importance of S-phase entry for efficient viral replication we artificially imposed a G0/G1 block prior to lytic reactivation. There are several commonly used methods of inducing cell-cycle arrest in cultured cells. However, we wanted to avoid compounds that directly interfere with DNA replication or that induce high levels of cell death in the absence of lytic induction. Serum depletion is a commonly used method for synchronizing cells prior to S-phase entry and effectively inhibited replication of viral genomes in this study. However, serum withdrawal negatively impacts cellular transcription, protein production and lipid-biosynthesis pathways which the virus may rely on to successfully execute the lytic programme. Consequently, to confirm that cells in G0/G1 do not support efficient viral replication, we turned to the CDK4/6 inhibitor palbociclib. This compound exerts anti-proliferative effects on phospho RB-positive tumour cells and has shown promise in treatment of advanced breast cancer [26]. In this investigation, CDK4/6 inhibition robustly restricted viral replication efficiency but also appeared to induce early lytic gene expression in the absence of doxycycline treatment. Despite this, it appears that cells treated with palbociclib alone do not support complete lytic replication of KSHV and display increased phosphorylation of H2AX indicating DNA damage. If this early lytic gene expression and DDR activation leads to increased cell death compared with uninfected cells then CDK4/6 inhibition could be a promising option as a targeted anti-viral agent. The potential of palbociclib to treat this aggressive B-cell lymphoma has been highlighted recently following the results of a screen examining drug-susceptible targets in PEL cells [27]. While the authors did not examine the lytic replication phase of KSHV, they did observe a robust G1 arrest in latently infected PEL cells as is presented here.

We have also demonstrated that phosphorylation of RPA32 and NBS1 following early viral gene expression is dependent on exit from G1. Previous experiments using ganciclovir, an inhibitor of viral DNA replication, showed that abrogation of late gene expression or viral genome amplification does not inhibit phosphorylation of RPA32 in PEL cells [10]. Together, these findings indicate that early lytic gene expression following KSHV reactivation specifically activates the DDR in S phase. Despite the impaired phosphorylation of DDR proteins following S-phase entry, we did not observe reduced phosphorylation of H2AX on serine 139 when lytic reactivation was limited to G1. Phosphorylated H2AX is a sensitive marker of DNA damage but can occur due to DNA fragmentation during apoptotic cell death [28]. The c-Jun N-terminal kinase can phosphorylate H2AX during programmed cell death and we did observe increased indicators of apoptosis in serum-depleted cells treated with doxycycline. However, the same increase in apoptosis was not evident following palbociclib treatment that also induced elevated levels of H2AX phosphorylation. It appears that while some aspects of the DDR are activated specifically in S phase, lytic replication in G1-arrested cells still promotes the appearance of this DNA damage marker.

Finally, we demonstrate here that KSHV reactivation causes increased frequency of stalled DNA replication forks. This increase was detected prior to amplification of viral DNA or late gene expression indicating that early lytic gene expression is sufficient to induce cellular replication stress. It has long been known that host DNA replication is halted in favour of viral genome amplification during the lytic stage of the herpesvirus lifecycle. While cell-cycle cessation in G1 has previously been postulated as a viral strategy to prevent cellular DNA replication, it was recently shown that the KSHV protein encoded by ORF59 interacts with the minichromosome maintenance protein complex (MCM), restricting its access to chromatin and hindering progression of replication forks [29]. While the authors did not measure the progression of individual replication forks using the fibre assay, the increase in stalled forks we have observed here could be the result of ORF59 expression or the combined actions of several viral proteins. Replication stress can lead to various types of DNA damage including long stretches of single-stranded DNA following helicase/polymerase uncoupling and one-sided DSBs following fork cleavage [30]. It is likely that viral-induced replication stress contributes to the S-phase-specific DDR activation observed after lytic induction and may also serve to halt cellular DNA replication and block cell-cycle progression to mitosis.

In summary, we have confirmed the importance of S-phase entry for productive KSHV lytic replication in B cells. Significantly, we have also demonstrated the effectiveness of the chemotherapeutic drug palbociclib against KSHV-infected B cells through both its restriction of cellular proliferation during latency and its deleterious effect on the production of infectious virions following lytic induction. Prognosis for patients with PEL remains poor with treatment options limited to standard chemotherapy regimens and relapse common after treatment [31]. The fact that lytic gene expression has been implicated in KSHV-mediated tumourigenesis, and is also required for dissemination of infectious virus in the tumour microenvironment [32], underlines the importance of understanding virus–host interactions that occur during this lifecycle phase. In turn, this will hopefully contribute to the development of new therapeutic approaches targeting aggressive virus-associated malignancies such as PEL.

## Methods

### Cell culture

BCBL-1 cells were cultured in RPMI media (Gibco) supplemented with 10 % FBS (Sigma-Aldrich) and 1 % penicillin-streptomycin (Gibco), while TREx BCBL-1-RTA cells were cultured in the same media with the addition of 100 µg ml^−1^ of hygromycin B (Roche). Latent KSHV in TREx BCBL-1-RTA cells was reactivated by addition of 0.5 µg ml^−1^ doxycycline (Sigma Aldrich). EA.hy926 cells were cultured in DMEM (Sigma Aldrich) supplemented with 10 % FBS and 1 % penicillin-streptomycin. All cell lines were cultured at 37 °C, 5 % CO_2_ in a humidified incubator. To induce cell-cycle arrest in G1, 1 µM palbociclib (PD-0332991) (Sigma Aldrich) was added to culture media.

### PI staining

To analyse cell-cycle distribution, TREx BCLB-1-RTA cells were washed in PBS, fixed in 70 % ethanol and stored at −20 °C. Fixed cells were washed in PBS twice prior to addition of PBS containing 20 µg ml^−1^ RNase (Sigma Aldrich) and 10 µg ml^−1^ propidium iodide (PI) solution (Sigma Aldrich). Relative DNA content was then analysed using an Accuri C6 flow cytometer (BD) and FlowJo software (Tree Star). Inhibition of viral DNA replication was achieved by addition of 100 µg ml^−1^ phosphonoacetic acid (PAA) (Sigma Aldrich) to culture media.

### Western blots

Whole-cell extracts were prepared by suspension of cell pellets in UTB buffer (8 M urea, 50 mM Tris HCl pH 7.4, 150 mM β-mercaptoethanol) followed by sonication on ice. Protein quantification was carried out using Bradford reagent (Bio-Rad), and appropriate volumes suspended in Laemmli sample buffer (Bio-Rad) before resolution of proteins using standard SDS-PAGE procedures.

The following primary antibodies were used for Western blotting in this study: K8α/K-bZIP (Sigma Aldrich, SAB5300152), ORF6/SSB (provided by Professor Gary Hayward), K8.1A (provided by Professor David Blackbourn). Cyclin A (Santa Cruz, sc-596), Cyclin E (Santa Cruz, sc-247), Cyclin B1 (Santa Cruz, sc-752), NBS1 (Genetex, GTX70222), phospho-NBS1 (S343) (Abcam, ab47272), RPA32 (Calbiochem, NA19L), phospho-RPA32 (S4/S8) (Bethyl, A300-245A), H2AX (Cell Signalling, 7631), γH2AX (S139) (Merck Millipore, 05–636), Histone H3 antibody (Abcam, ab18521), Phospho-Histone H3 (S10) (Cell Signalling, 9701), β-Actin (Sigma, A2228). Proteins were then detected using polyclonal Goat Anti-Mouse (Dako Laboratories, P0447) or polyclonal Swine Anti-Rabbit (Dako Laboratories, P0399) secondary antibodies conjugated to HRP. Proteins were detected using enhanced chemiluminescence (ECL) reagent (GE Healthcare) and autoradiography. Densitometry of cyclin blots was carried using ImageJ software (National Institutes of Health; http://rsbweb.nih.gov/ij/) with values normalized to a β-Actin loading control.

### Immunofluorescence microscopy

BCBL-1 cells were cytospun onto glass slides, fixed in 4 % paraformaldehyde and permeabilized in 0.5 % Triton X-100 (Sigma Aldrich). Cells were blocked in 10 % heat inactivated goat serum (HINGS) for 30 min followed by addition of primary antibodies diluted in 10 % HINGS for 1 h. Slides were washed three times in PBS followed by addition of anti-rabbit Alexa Fluor 594 and anti-mouse Alexa Fluor 488 conjugated secondary antibodies (Thermo Fisher Scientific) for 1 h. Following three washes in PBS, slides were counterstained with DAPI nuclear stain (Life Technologies) and coverslips mounted with Prolong Gold antifade reagent (Life Technologies). The following primary antibodies were used for immunofluorescence: ORF6/SSB (provided by Professor Gary Hayward), Mitosin (Abcam, ab90). Relative mitosin intensity corrected for background fluorescence was calculated using ImageJ software on 150 cells from each condition across three independent experiments.

Quantification of infectious virus production from TREx BCBL-1-RTA was carried out as previously described [11]. In brief, supernatants containing infectious KSHV were added to EA.hy926 cells grown on coverslips and spinoculated (330 ***g***, 20 min, room temperature). After 48 h, the percentage of infected cells was determined by immunofluorescence staining for LANA (Novacastra). For each independent experiment, a minimum of 500 cells were analysed and statistical analysis was performed using GraphPad Prism (GraphPad Software).

### Quantitative PCR

Determination of relative viral load was carried out as previously described [11]. Briefly, genomic DNA was extracted from TREx BCBL-1-RTA cells using a Wizard genomic DNA purification kit (Promega) and amplification reactions were performed in an Applied Biosystems 7500 Fast Dx real-time PCR machine. Primers were used against the cellular β2-microglobulin (B2m) gene and the viral ORF47 gene. Viral load in induced cells was calculated as fold change relative to uninduced controls using the comparative threshold cycle (C_T_) method.

The following primer sequences were used B2m-Fwd (5′-GGAATTGATTTGGGAGAGCATC-3′) and B2m-Rev, (5′-CAGGTCCTGGCTCTACAATTTACTAA-3′), and viral DNA; ORF47-Fwd (5′-CGCGGTCGTTCGAAGATTGGG-3′) and ORF47-Rev (5′-CGAGTCTGACTTCCGCTAACA-3′).

### DNA fibre analysis

Sub-confluent TREx BCBL-1-RTA cells were treated with 0.5 µg ml^−1^ doxycycline for 10 h before chloro-deoxyuridine (CldU) (Sigma Aldrich), at a final concentration of 25 µM, was added to cell-culture media for 20 mins. Cells were pelleted and washed in media containing 250 µM iodo-deoxyuridine (IdU) (Sigma Aldrich) before being re-suspended in media containing 250 µM IdU for a total exposure to IdU of 20 mins. Cells were pelleted and re-suspended in ice cold PBS at a concentration of 5×10^5^ ml^−1^. 2 µl of cell suspension was placed on glass slides, mixed with 7 µl of spreading buffer (200 mM Tris-HCl, pH 7.5, 50 mM EDTA, 0.5 % SDS) and spread across the glass by tilting at an angle. Slides were air dried and fixed in 3 : 1 methanol, acetic acid for 10 mins. For immunostaining of DNA fibres, DNA was denatured using 2.5 M HCL for 1 h, washed in PBS and submerged in blocking solution (1 % BSA, 1 % Tween 20 in PBS) for 30 mins. DNA immunostaining was carried out by submersion in rat anti-BrdU (clone BU1/75, ICR1; Abcam, ab6326), and mouse anti-BrdU (clone B44; BD Biosciences, 347583) in blocking buffer for 1 h. Following three washes in PBS-Tween followed by incubation with anti-rat Alexa Fluor 555 and anti-mouse Alexa Fluor 488 (Thermo Fisher Scientific). DNA fibres were visualised by immunofluorescence microscopy using a Leica DM6000B epifluorescence microscope.
